# Superior response and survival of intensive chemotherapy over venetoclax plus azacitidine in newly diagnosed KIT-mutated acute myeloid leukemia

**DOI:** 10.1007/s00277-026-06841-4

**Published:** 2026-02-16

**Authors:** Qingli Ji, Xinwen Jiang, Xiaoqing Li, Chen Cao, Xinrui Zhang, Minran Zhou, Sai Ma, Chunyan Chen

**Affiliations:** Department of Hematology, Qilu Hospital, Shandong University, No. 107 Wenhuaxi Road, Jinan, 250012 China

**Keywords:** Acute myeloid leukemia, KIT, Intensive chemotherapy, Venetoclax

## Abstract

Although KIT mutations hold significant prognostic value in acute myeloid leukemia (AML), their impact on selecting first-line treatment remains unclear. This retrospective study of 222 newly diagnosed AML patients therefore compared the efficacy of venetoclax plus azacitidine (VA) versus intensive chemotherapy (IC) in KIT-mutated AML, while also exploring the prognostic implications of KIT mutation subtypes and their role in predicting VA response. Among patients with KIT mutations, IC was superior to VA, yielding significantly longer median event-free survival (EFS) (14.5 vs. 2.4 months, *p* = 0.011) and overall survival (OS) (not reached vs. 9.8 months, *p* < 0.0001), and a higher complete remission (CR) rate (80.0% vs. 17.6%, *p* < 0.001). Exon 17 mutations were associated with significantly shorter EFS relative to other KIT mutations (7.3 vs. 18.8 months; *p* = 0.046). Moreover, among all VA-treated patients, KIT mutation was an independent adverse prognostic factor for both EFS (HR = 3.25, *p* < 0.001) and OS (HR = 3.31, *p* = 0.001). This study demonstrates the potential superiority of IC over VA in KIT-mutated AML and identifies KIT mutation as an important biomarker of resistance to venetoclax-based therapy, providing valuable guidance for first-line treatment decisions.

**Supplementary Information:**

The online version contains supplementary material available at 10.1007/s00277-026-06841-4.

## Introduction

Acute myeloid leukemia (AML) is a highly heterogeneous hematologic malignancy. Molecular genetic abnormalities play a pivotal role in guiding disease classification, risk stratification, and treatment selection. *KIT* gene mutations represent an important driver mutation in AML. Although their overall frequency in AML is relatively low, *KIT* mutations occur in 20–40% of core-binding factor AML (CBF-AML) and are associated with an increased risk of relapse and poorer prognosis [1–3]. Traditionally, for patients considered medically fit, typically characterized by younger age, good performance status, and limited comorbidities, intensive chemotherapy (IC) was the standard first-line treatment.

In recent years, low-intensity regimens such as venetoclax combined with azacitidine (VA) have demonstrated remarkable efficacy in untreated AML patients unfit for intensive induction therapy, establishing a cornerstone of first-line therapy for this population [4–6]. However, the differential responses of *KIT*-mutated AML patients to VA versus IC, and the underlying molecular mechanisms, remain inadequately defined. Notably, *KIT* mutations exhibit significant heterogeneity; exon 17 mutations (particularly D816V), the most common subtype, may confer distinct biological properties and clinical implications [7–10]. Consequently, elucidating the impact of *KIT* mutation status and its subtypes on the efficacy of first-line treatment regimens has emerged as a critical, unresolved clinical question.

This study aims to conduct a retrospective cohort analysis to systematically compare the efficacy and safety of the VA versus IC as first-line therapy in *KIT*-mutated AML patients. Furthermore, it seeks to investigate the potential influence of different *KIT* mutation subtypes, especially exon 17 mutations, on treatment response. By integrating genetic mutational profiles, treatment responses, survival outcomes, clonal dynamic monitoring, and multivariate analyses, we endeavor to provide novel evidence-based insights for personalizing treatment strategies in *KIT*-mutated AML patients.

## Methods

### Study design and patient cohorts

This retrospective study enrolled consecutive newly diagnosed AML patients at Qilu Hospital of Shandong University from January 2017 to January 2025. Inclusion criteria were: age ≥ 18 years, newly diagnosed AML, available *KIT* gene testing data, and complete clinical data. Exclusion criteria were: acute promyelocytic leukemia, secondary AML, not receiving first-line IC or VA treatment, missing key data, and loss to follow-up.

A total of 222 patients were included. For the core analysis, patients were stratified into three cohorts based on *KIT* mutation status and first-line treatment: *KIT*-mutated/VA (Cohort A, *n* = 17), *KIT*-mutated/IC (Cohort B, *n* = 50), and *KIT*-wild-type/VA (Cohort C, *n* = 155). Cohort C, which consisted exclusively of *KIT*-wild-type patients who received first-line VA, was established to provide a uniform treatment comparator for assessing the prognostic impact of *KIT* mutations within the VA-treated population. Furthermore, to analyze the impact of mutation subtypes, all *KIT*-mutated patients (*n* = 67) were categorized into exon 17 mutation (Cohort D, *n* = 53) and non-exon 17 mutation (Cohort E, *n* = 14) subgroups.

The study protocol was performed in accordance with the Declaration of Helsinki and was approved by the Ethics Committee of Qilu Hospital of Shandong University.

## Data collection and molecular profiling

Baseline clinical and laboratory data, including cytogenetic and molecular genetic features, were collected from the hospital’s electronic medical record system. *KIT* and other gene mutations were detected by next-generation sequencing (NGS). *KIT* sequencing covered all exons, with deep sequencing of exon 17 hotspots (including D816V, N822K, etc.) at 1% sensitivity. The comprehensive NGS panel interrogated 72 genes for DNA sequencing and 54 genes for RNA fusion detection (Table S1, Online Resource 1).

## Treatment and risk stratification

All treatment regimens were determined based on patient age, Eastern Cooperative Oncology Group performance status (ECOG PS), comorbidities, and physician discretion. For patients under 65 years with good performance status (ECOG 0–1), the selection of VA over IC was typically based on a comprehensive assessment that included specific comorbidities not fully reflected by the performance status score (e.g., uncontrolled cardiac dysfunction, severe hepatic or renal impairment, active infection), as well as patient preference. IC was primarily the standard “7 + 3” protocol, involving an anthracycline (e.g., idarubicin or daunorubicin) combined with cytarabine. VA consisted of azacitidine administered for 7 days per cycle with a standard venetoclax dose ramp-up to the target dose. A small number of *KIT*-mutated patients subsequently received the *KIT* inhibitor avapritinib. All patients were risk-stratified according to the European LeukemiaNet (ELN) 2022 recommendations.

## Efficacy and safety assessments

Treatment responses were evaluated per ELN 2022 criteria. Complete remission (CR) required < 5% marrow blasts, no extramedullary disease, and count recovery; CR with incomplete hematologic recovery (CRi) required all CR criteria except cytopenias; morphologic leukemia-free state (MLFS) required < 5% blasts without count recovery. Overall response rate (ORR) included CR + CRi + MLFS. Minimal residual disease (MRD) negativity was defined as < 0.1% leukemic cells by flow cytometry post-induction. Overall survival (OS) was from diagnosis to death from any cause or last follow-up; event-free survival (EFS) was from treatment start to treatment failure, relapse, or death from any cause. Early mortality included deaths within 2 months; adverse events were graded by Common Terminology Criteria for Adverse Events (CTCAE) v5.0. Additional evaluations included *KIT* mutation clearance kinetics (at 3, 6, and 12 months), the impact of allogeneic hematopoietic stem cell transplantation (allo-HSCT) on prognosis, and co-mutation patterns on survival.

### Statistical analysis

All statistical analyses were performed using R software (version 4.4.2). Categorical variables were compared using Chi-square or Fisher’s exact tests; continuous variables using Mann-Whitney U or Kruskal-Wallis tests. Survival was analyzed by Kaplan-Meier method with log-rank test. Univariate Cox regression (*P* < 0.15) identified variables for multivariate Cox models. Results are expressed as hazard ratios (HR) with 95% confidence intervals (CI). Propensity score matching (PSM) (1:1, caliper = 0.2) controlled confounding. Transplantation impact was assessed via time-dependent Cox regression. Mutation profiles were visualized using maftools. All tests were two-sided; *P* < 0.05 was considered significant.

## Results

### Patient baseline characteristics

**Fig. 1:**
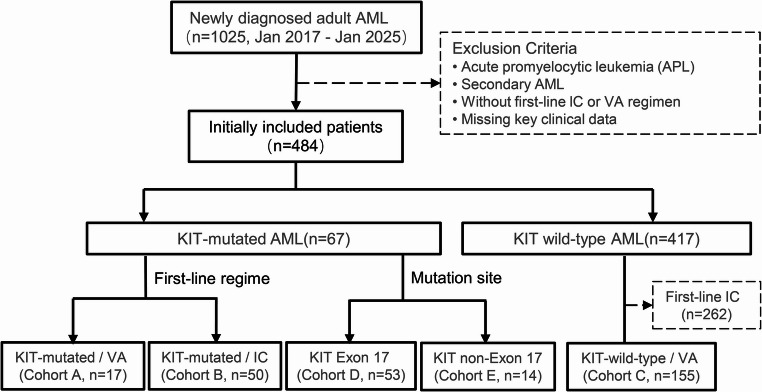
Study flow diagram of patient selection and cohort stratification

**Fig. 2:**
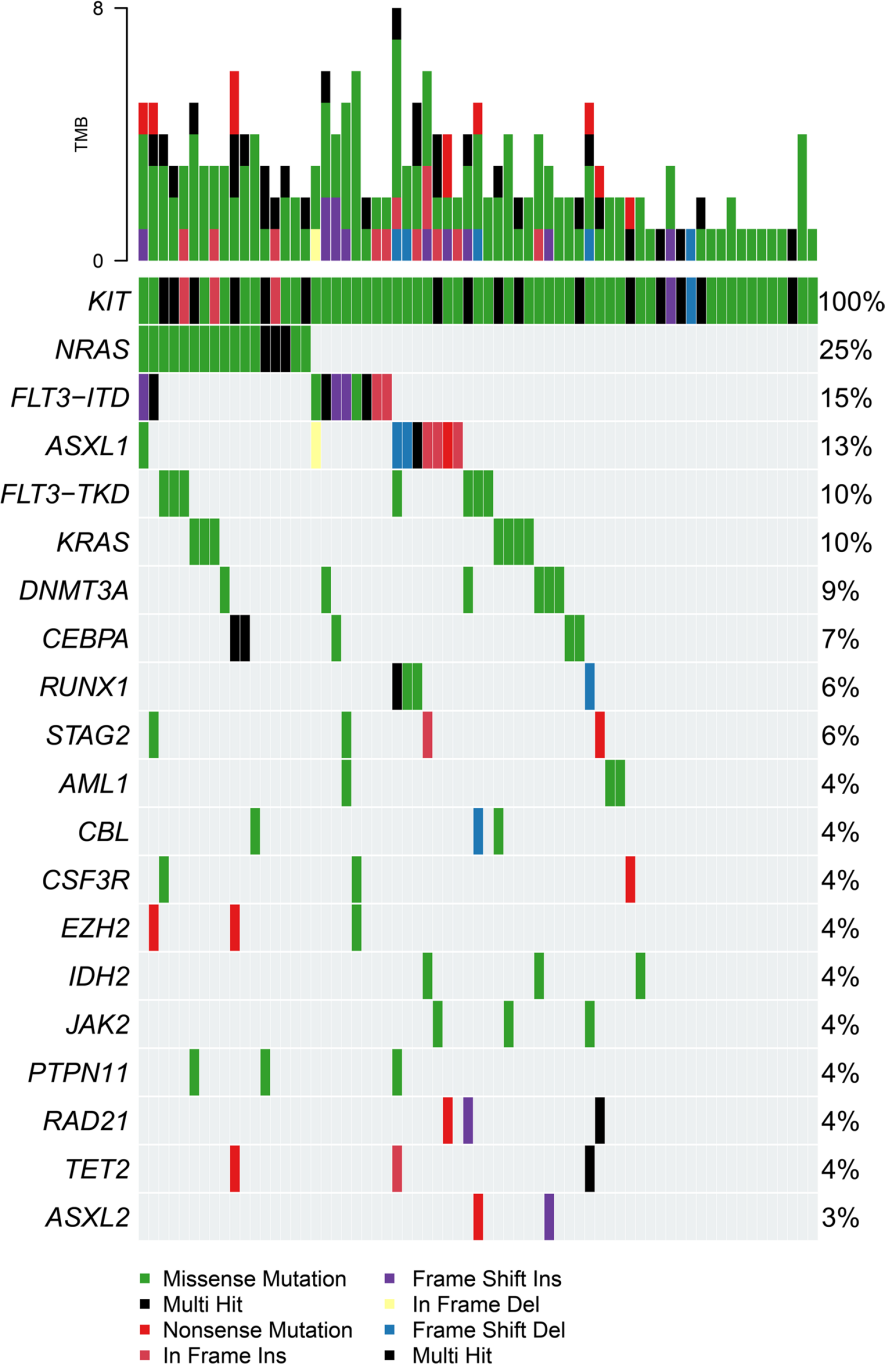
Genetic Landscape of KIT-Mutated AML

As detailed in Fig. 1, a total of 222 newly diagnosed AML patients were included in the final analysis. Baseline characteristics are summarized in Table 1. Compared with Cohort C (*KIT*-wild-type/VA), patients in Cohort A (*KIT*-mutated/VA) had a significantly higher proportion of age < 65 years (94.1% vs. 51.0%), Eastern Cooperative Oncology Group (ECOG) performance status ≥ 2 (58.8% vs. 20.6%), and French-American-British (FAB) M2 subtype (64.7% vs. 24.5%). Other baseline features were largely balanced across comparison groups. In *KIT*-mutated AML patients, the co-mutation landscape (Fig. 2) identified NRAS (25%), FLT3-ITD (15%), and ASXL1 (13%) as the most frequent co-occurring mutations. *KIT* mutations were primarily missense variants, overwhelmingly localized to exon 17 (79.10%) and dominated by the D816V allele (66.04%); other recurrent exon 17 mutations included N822K, D816Y, and D816H (Table S2, Online Resource 1).

A total of 222 newly diagnosed AML patients were included in the final analysis and categorized into three primary cohorts(A-C) based on KIT mutation status and first-line treatment. Furthermore, all KIT-mutated patients were stratified into two subgroups (D and E) by mutation site (exon 17 vs. non-exon 17) for subsequent analysis.

AML, acute myeloid leukemia; APL, acute promyelocytic leukemia; IC, intensive chemotherapy; VA, venetoclax plus azacitidine.

**Table 1:** Baseline characteristics of the study cohorts

Characteristics	KIT ^mut^ / VA (Cohort A, *n* = 17)	KIT ^mut^ / IC (Cohort B, *n* = 50)	KIT ^wt^ / VA (Cohort C, *n* = 155)	*p* (A vs. B)	*p* (A vs. C)	Exon17 (Cohort D, *n* = 53)	Non-exon17(Cohort E, *n* = 14)	*p* (D vs. E)
**Age**				1.000	0.002**			1.000
< 65 years	16 (94.1%)	48 (96%)	79 (51%)			50 (94.3%)	14 (100%)	
≥ 65 years	1 (5.9%)	2 (4%)	76 (49%)			3 (5.7%)	0 (0%)	
**Gender**				0.917	0.674			0.751
Male	11 (64.7%)	35 (70%)	87 (56.1%)			37 (69.8%)	9 (64.3%)	
Female	6 (35.3%)	15 (30%)	68 (43.9%)			16 (30.2%)	5 (35.7%)	
**ECOG PS**				0.928	0.001**			1.000
0–1	7 (41.2%)	18 (36%)	123 (79.4%)			20 (37.7%)	5 (35.7%)	
≥ 2	10 (58.8%)	32 (64%)	32 (20.6%)			33 (62.3%)	9 (64.3%)	
**FAB**				0.019*	0.004**			0.479
M2	11 (64.7%)	14 (28%)	38 (24.5%)			20 (37.7%)	5 (35.7%)	
M5	5 (29.4%)	20 (40%)	92 (59.4%)			18 (34%)	7 (50%)	
Other	1 (5.9%)	16 (32%)	25 (16.1%)			15 (28.3%)	2 (14.3%)	
**ELN 2022 risk**				0.330	0.098			< 0.001***
Favorable	4 (23.5%)	17 (34%)	34 (21.9%)			9 (17%)	12 (85.7%)	
Intermediate	9 (52.9%)	28 (56%)	47 (30.3%)			36 (67.9%)	1 (7.1%)	
Adverse	4 (23.5%)	5 (10%)	74 (47.7%)			8 (15.1%)	1 (7.1%)	
**WBC (×10⁹/L)**	10.9 (4.6–39.5)	18.1 (4.5–37.9)	7 (2.4–28.5)	0.676	0.307	13.4 (5-38.2)	15 (3.5–40.5)	0.994
**HGB (g/L)**	68 (56–97)	82 (65-103.8)	75 (65–87)	0.210	0.417	80 (65–105)	65.5 (53.8–84.8)	0.081
**PLT (×10⁹/L)**	19 (14–34)	36.5 (24–63)	38 (24–77)	0.015*	0.002**	32 (19–60)	30.5 (20-52.2)	0.926
**BM blast**				0.682	0.404			0.672
< 40%	3 (17.6%)	6 (12%)	45 (29%)			8 (15.1%)	1 (7.1%)	
≥ 40%	14 (82.4%)	44 (88%)	110 (71%)			45 (84.9%)	13 (92.9%)	
**WHO 2022 Classification**				0.931	< 0.001***			0.021*
AML with RUNX1::RUNX1T1	10 (58.8%)	26 (52%)	2 (1.3%)			32 (60.4%)	4 (28.6%)	
AML with CBFB::MYH11	3 (17.6%)	12 (24%)	4 (2.6%)			8 (15.1%)	7 (50%)	
Other	4 (23.5%)	12 (24%)	149 (96.1%)			13 (24.5%)	3 (21.4%)	
**Complex karyotype**	0 (0%)	2 (4%)	29 (18.7%)	1.000	0.080	2 (3.8%)	0 (0%)	1.000
**DNMT3A**	3 (17.6%)	3 (6%)	50 (32.3%)	0.166	0.336	6 (11.3%)	0 (0%)	0.330
**NPM1**	0 (0%)	2 (4%)	38 (24.5%)	1.000	0.015*	2 (3.8%)	0 (0%)	1.000
**TET2**	0 (0%)	3 (6%)	35 (22.6%)	0.565	0.025*	3 (5.7%)	0 (0%)	1.000
**NRAS**	3 (17.6%)	14 (28%)	34 (21.9%)	0.527	1.000	10 (18.9%)	7 (50%)	0.034*
**FLT3-ITD**	1 (5.9%)	9 (18%)	33 (21.3%)	0.432	0.200	10 (18.9%)	0 (0%)	0.106
**TP53**	0 (0%)	0 (0%)	11 (7.1%)	-	-	0 (0%)	0 (0%)	-
**Allo-HSCT**	1 (5.9%)	12 (24%)	10 (6.5%)	0.158	1.000	9 (17%)	4 (28.6%)	0.447

*ECOG PS* Eastern Cooperative Oncology Group Performance Status, *FAB* French-American-British classification, *ELN* European LeukemiaNet, *WBC* white blood cell count, *HGB* hemoglobin, *PLT* platelet count, *BM* bone marrow, *Allo-HSCT* allogeneic hematopoietic stem cell transplantation, *KIT*^*mut*^ KIT-mutated, *KIT*^*wt*^ KIT wild-type, *VA* venetoclax plus azacitidine, *IC* intensive chemotherapy

Data presentation: Categorical, n (%); continuous, median (IQR)

*p < 0.05, **p < 0.01, ***p < 0.001

Oncoprint displaying the spectrum of frequent gene mutations in 67 KIT-mutated AML patients. The profile shows that KIT mutations are predominantly missense variants. The most frequently co-occurring mutations were in NRAS (25%), FLT3-ITD (15%), and ASXL1 (13%).

### Efficacy and Survival Comparison of IC versus VA in *KIT*-Mutated Patients

**Fig. 3:**
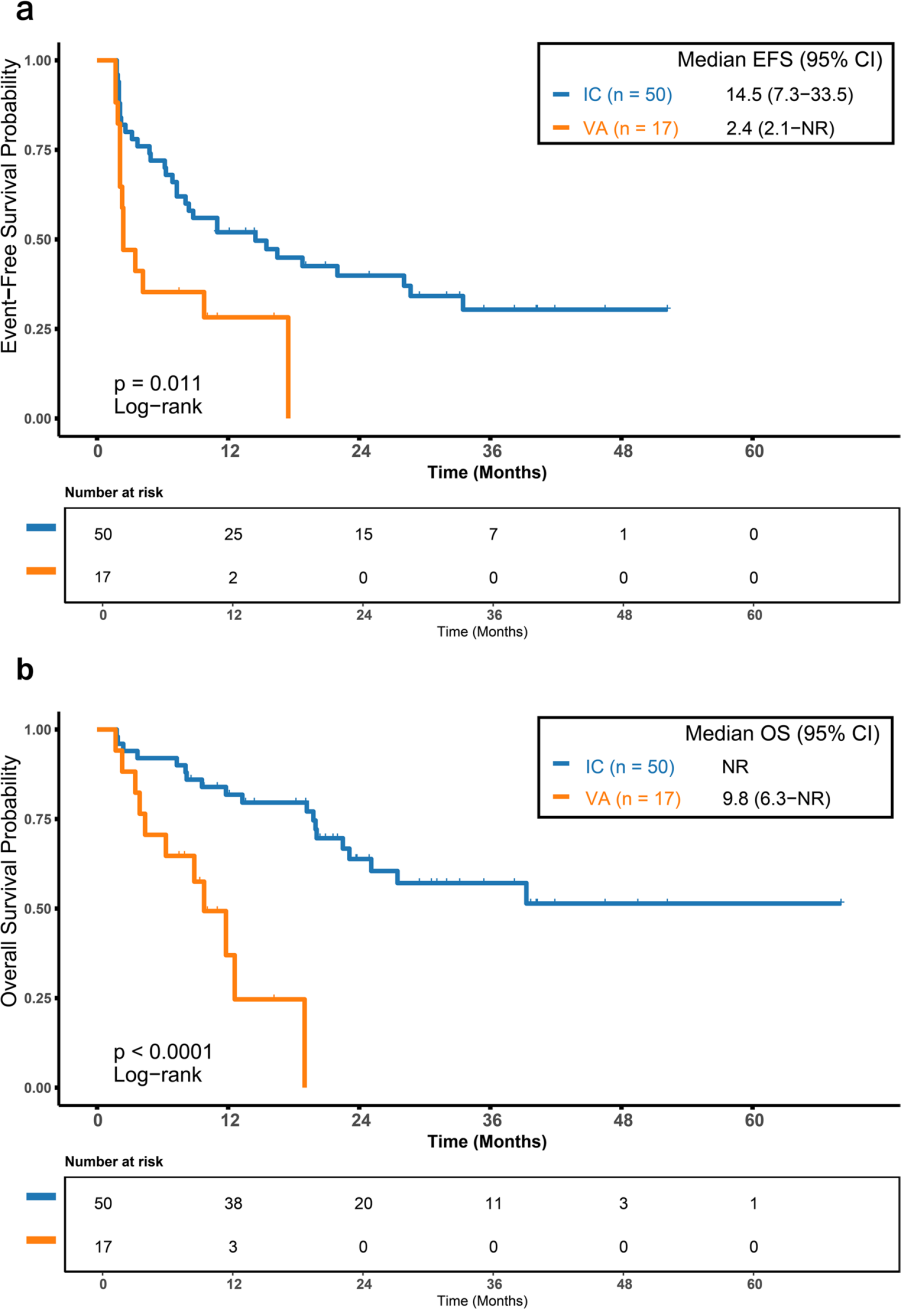
Survival Outcomes of First-Line Therapy in KIT-Mutated AML (**a**) Event-free survival (EFS)and (**b**) overall survival (OS) in KIT-mutated patients receiving first-line intensive chemotherapy (IC) versus venetoclax plus azacitidine (VA). The IC cohort demonstrated significantly longer median EFS (14.5 vs. 2.4 months; *p* = 0.011) and OS (not reached vs. 9.8 months; *p* < 0.0001). EFS, event-free survival; OS, overall survival; IC, intensive chemotherapy; VA, venetoclax plus azacitidine; NR, not reached

 We first compared the survival outcomes of *KIT*-mutated AML patients receiving first-line IC versus those receiving VA. The IC cohort demonstrated significantly longer median EFS (14.5 vs. 2.4 months, *p* = 0.011; Fig. 3a) and OS (not reached vs. 9.8 months, *p* < 0.0001; Fig. 3b) compared to the VA cohort. To control for potential confounding bias, PSM was performed, yielding 12 matched pairs. After matching, the IC cohort maintained numerical advantages in median EFS (11.0 vs. 3.0 months, *p* = 0.360; Figure S1a, Online Resource 1) and median OS (not reached vs. 11.8 months, *p* = 0.071; Figure S1b, Online Resource 1), although these differences were not statistically significant.

We next compared treatment responses and safety between the IC and VA cohorts (Fig. 5a). The IC cohort achieved significantly higher CR (80.0% vs. 17.6%, *p* < 0.0001), ORR (82.0% vs. 41.2%, *p* < 0.01), and MRD-negative (76.0% vs. 35.3%, *p* < 0.01) rates compared to the VA cohort. Regarding safety, the VA cohort showed higher incidences of grade ≥ 3 adverse events (82.4% vs. 72.0%, *p* = 0.527) and early death (5.9% vs. 2.0%, *p* = 0.446), though these differences were not statistically significant.

**Fig. 4:**
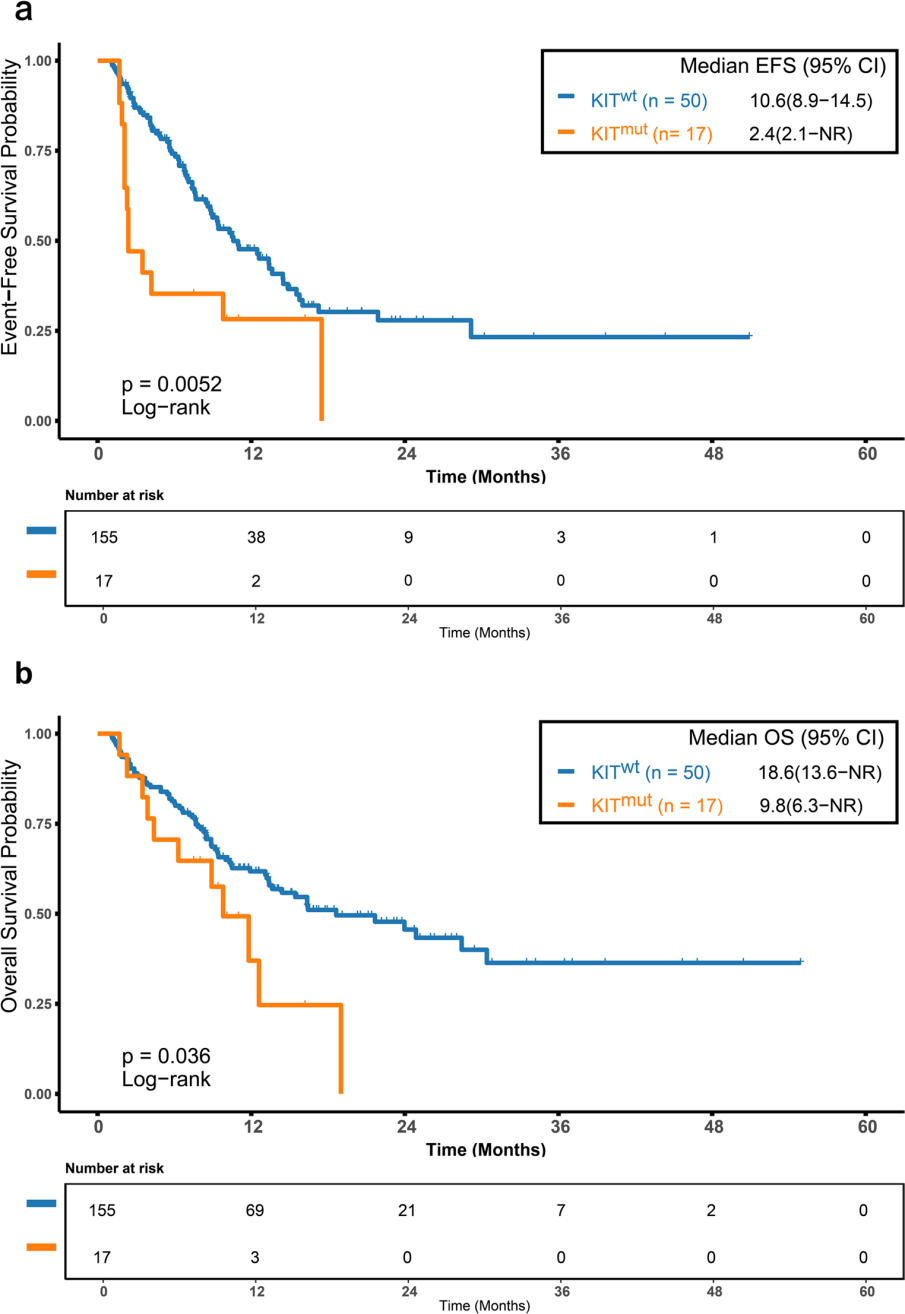
Prognostic Impact of KIT Mutation in VA-Treated Patients. (**a**) Event-free survival (EFS)and (**b**) overall survival (OS) in VA-treated patients with KIT mutation versus KIT wild-type. Patients with KIT mutations had significantly shorter median EFS (2.4 vs. 10.6 months; *p* = 0.005) and OS (9.8 vs. 18.6 months; *p* = 0.036). EFS, event-free survival; OS, overall survival; KIT^mut^, KIT-mutated; KIT^wt^, KIT wild-type; NR, not reached

### Impact of *KIT* Mutation Status on Efficacy and Prognosis in VA-Treated Patients

 We then assessed the prognostic impact of *KIT* mutations in the VA-treated population. Patients with *KIT* mutations had significantly shorter median EFS (2.4 vs. 10.6 months, *p* = 0.005; Fig. 4a) and OS (9.8 vs. 18.6 months, *p* = 0.036; Fig. 4b) than wild-type patients. PSM created 28 balanced pairs, and subsequent analysis showed that differences in EFS (14.5 vs. 13.4 months, *p* = 0.500; Figure S2a, Online Resource 1) and OS (39.3 months vs. not reached, *p* = 0.890; Figure S2b, Online Resource 1) were no longer significant. The attenuation of statistical significance is likely due to the reduced sample size after matching.

**Fig. 5:**
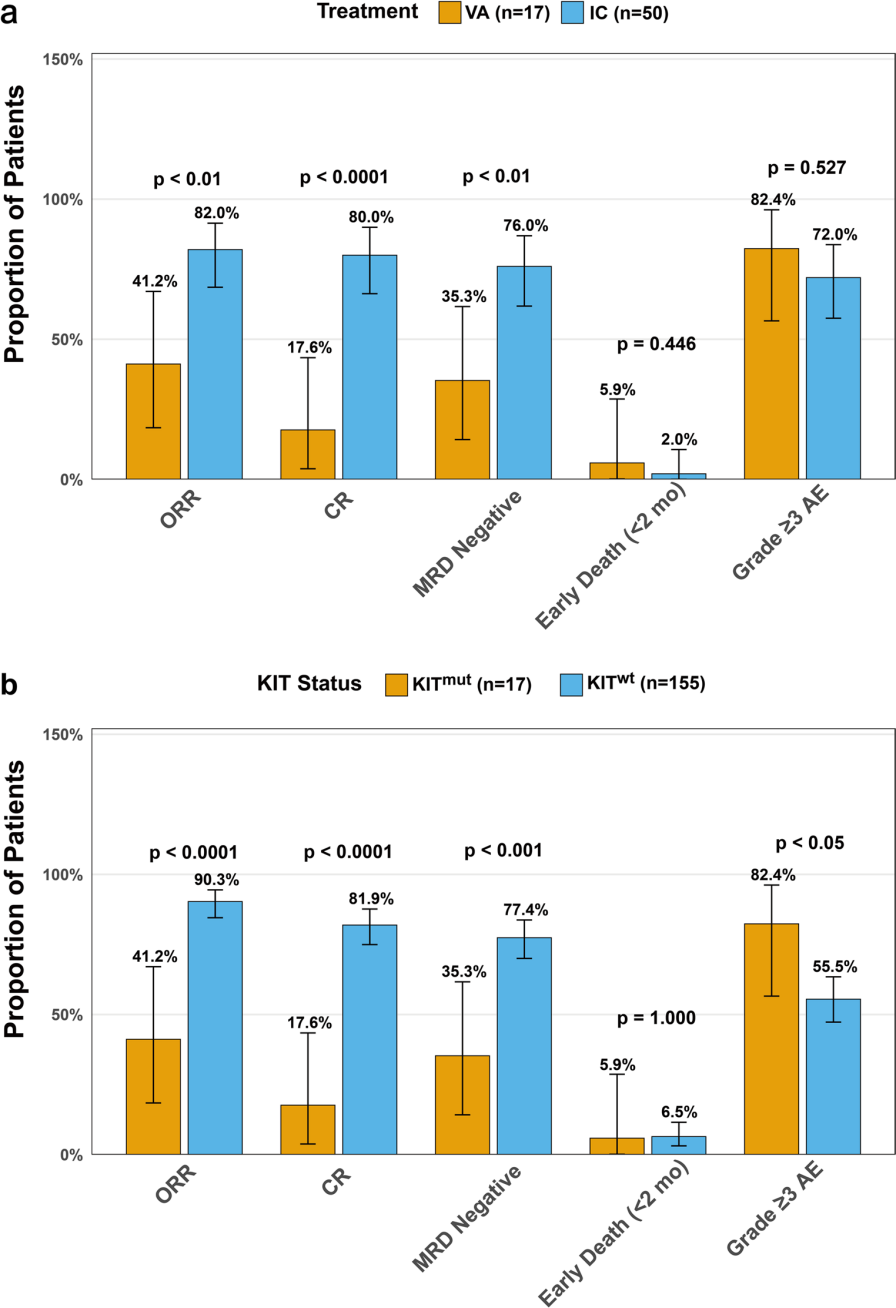
Comparison of Efficacy and Safety. (**a**) Bar graph showing rates of complete remission (CR), overall response rate (ORR), minimal residual disease (MRD) negativity, early death (< 2 months), and grade ≥ 3 adverse events (AEs) in KIT-mutated patients receiving first-line IC versus VA. The IC cohort achieved significantly higher CR (80.0% vs. 17.6%; *p* < 0.0001), ORR (82.0% vs. 41.2%; *p* < 0.01), and MRD-negative rates (76.0% vs. 35.3%; *p* < 0.01). Incidences of early death (2.0% vs. 5.9%; *p* = 0.446) and grade ≥ 3 AEs (72.0% vs. 82.4%; *p* = 0.527) were not significantly different. (**b**) Corresponding outcomes in VA-treated patients with KIT mutation versus KIT wild-type. The KIT-wild-type group had significantly higher CR (81.9% vs. 17.6%; *p* < 0.0001), ORR (90.3% vs. 41.2%; *p* < 0.0001), and MRD-negative rates (77.4% vs. 35.3%; *p* < 0.001), while also exhibiting a lower incidence of grade ≥ 3 AEs (55.5% vs. 82.4%; *p* < 0.05). Early death rates were similar (6.5% vs. 5.9%; *p* = 1.000). IC, intensive chemotherapy; VA, venetoclax plus azacitidine; ORR, overall response rate; CR, complete remission; MRD, minimal residual disease; AEs, adverse events; KIT^mut^, KIT-mutated; KIT^wt^, KIT wild-type

Regarding treatment response and safety (Fig. 5b), *KIT*-wild-type patients demonstrated significantly higher rates of CR (81.9% vs. 17.6%; *p* < 0.0001), ORR (90.3% vs. 41.2%; *p* < 0.0001), and MRD negativity (77.4% vs. 35.3%; *p* < 0.001) compared to *KIT*-mutated patients. Early death rates were similar between groups (6.5% vs. 5.9%; *p* = 1.000), but grade ≥ 3 adverse events occurred more frequently in the mutated cohort (82.4% vs. 55.5%; *p* < 0.050).

### Prognostic Impact of *KIT* Mutation Subtypes and Specific Co-mutations

**Fig. 6:**
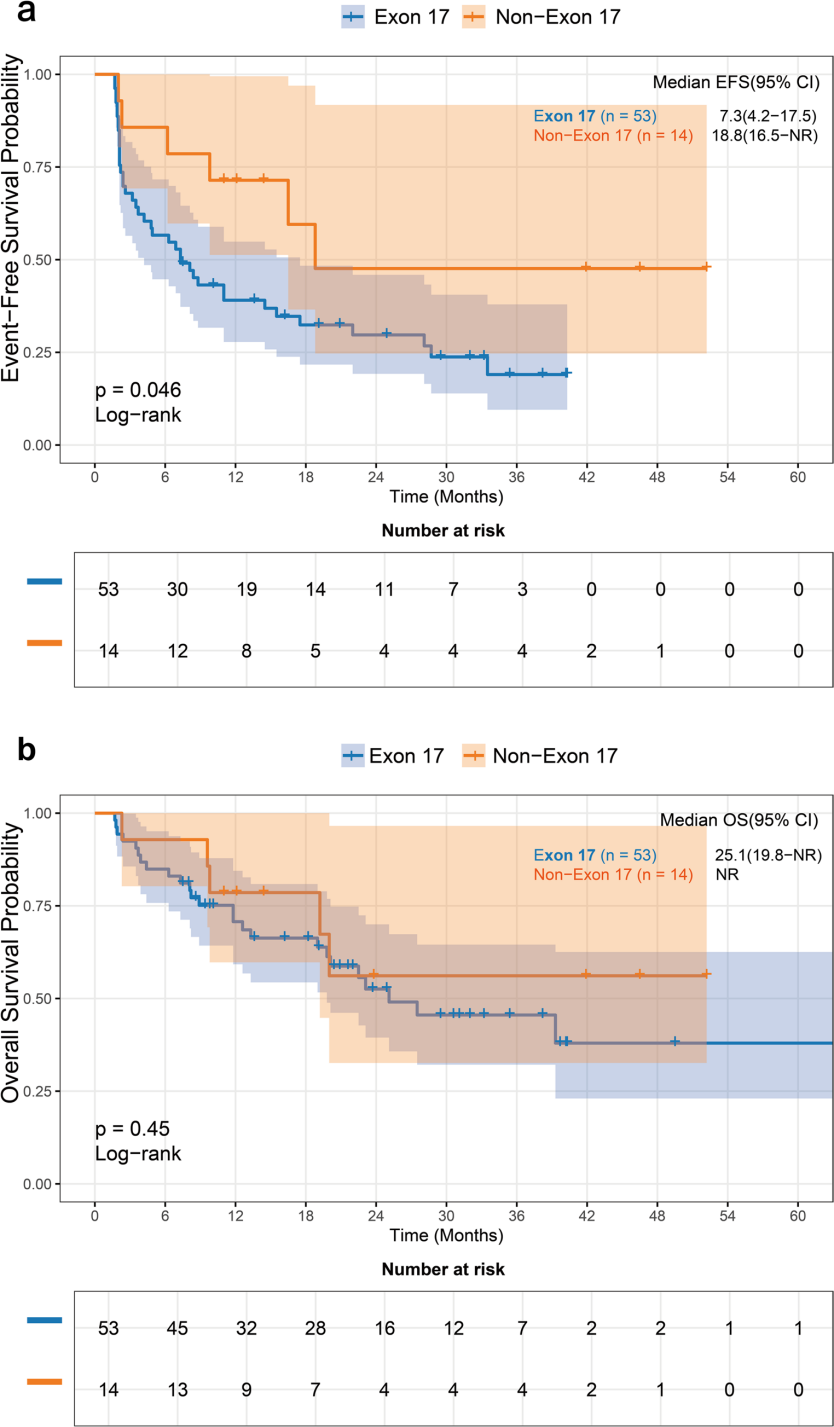
Impact of KIT mutation subtypes on survival. (**a**) Event-free survival (EFS) and (**b**) overall survival (OS) of KIT-mutated patients stratified by mutation site (exon 17 vs. non-exon 17). Patients harboring exon 17 mutations had significantly inferior median EFS compared to those with KIT mutations outside of exon 17 (7.3 vs. 18.8 months; *p* = 0.046). The difference in median OS (25.1 months vs. not reached; *p* = 0.450) was not statistically significant. EFS, event-free survival; OS, overall survival; NR, not reached.

 Among *KIT*-mutated patients, the exon 17 mutation group had significantly shorter median EFS than the non-exon 17 mutation group (7.3 vs. 18.8 months, *p* = 0.046; Fig. 6a). However, the difference in OS (25.1 months vs. not reached, *p* = 0.450; Fig. 6b) was not statistically significant.

Co-mutation analysis revealed that *KIT*-mutated patients with concurrent *NRAS* mutations had significantly longer EFS (not reached vs. 7.6 months, *p* = 0.003; Figure S3a, Online Resource 1) and OS (not reached vs. 20.1 months, *p* = 0.004; Figure S3b, Online Resource 1) compared to *SNRAS*-wild-type patients. In contrast, those with *FLT3-ITD/TKD* mutations showed lower median EFS (7.3 vs. 16.5 months, *p* = 0.220; Figure S4a, Online Resource 1) and median OS (22.5 vs. 39.3 months, *p* = 0.470; Figure S4b, Online Resource 1) compared to *FLT3*-wild-type patients, but these differences were not significant.

## Multivariate analysis of prognostic factors in VA-Treated patients

**Fig. 7:**
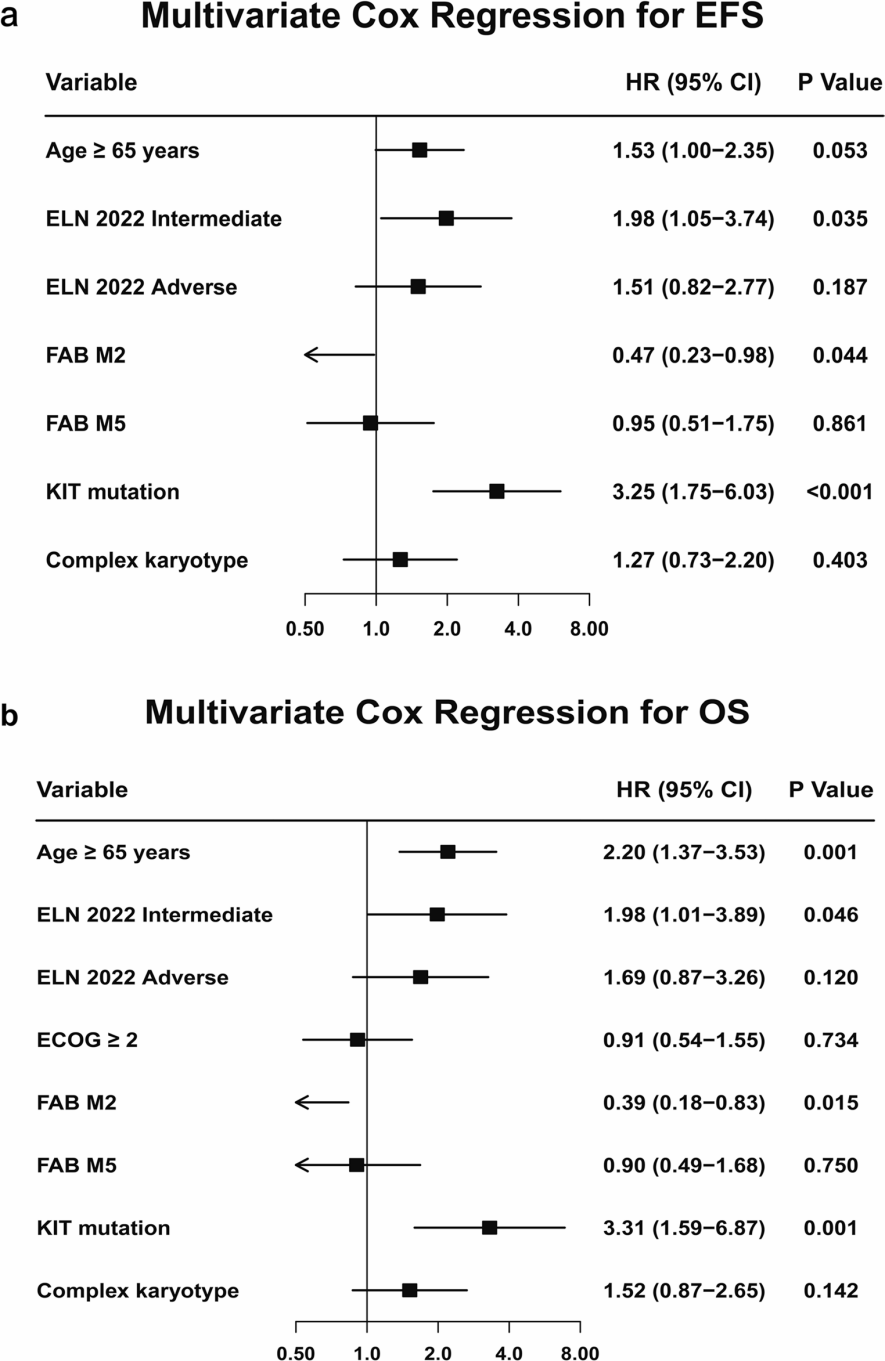
Multivariate Analysis of Prognostic Factors in VA-Treated Patients. (**a**) Factors associated with event-free survival (EFS). (**b**) Factors associated with overall survival (OS)

Multivariate Cox analysis confirmed *KIT* mutation as an independent adverse prognostic factor for both EFS (HR = 3.25, 95% CI 1.75–6.03, *p* < 0.001; Fig. 7a) and OS (HR = 3.31, 95% CI 1.59–6.87, *p* = 0.001; Fig. 7b) in VA-treated patients. Furthermore, the analysis showed that age ≥ 65 years was a risk factor for OS (HR = 2.20, 95% CI 1.37–3.53, *p* < 0.001), whereas the FAB-M2 subtype predicted better EFS (HR = 0.47, 95% CI 0.23–0.98, *p* = 0.044) and OS (HR = 0.39, 95% CI 0.18–0.83, *p* = 0.015). To ensure the robustness of our findings within the latest risk framework for venetoclax-based therapy, we also stratified the VA-treated cohort according to the ELN 2024 risk classification. In this model, KIT mutation remained an independent adverse prognostic factor for both EFS (HR = 3.30, 95% CI 1.76–6.19, *p* < 0.001) and OS (HR = 3.52, 95% CI 1.68–7.39, *p* < 0.001) (Figure S5, Online Resource 1).

Forest plots showing factors independently associated with(a) event-free survival (EFS) and (b) overall survival (OS) in patients treated with venetoclax plus azacitidine (VA). KIT mutation was an independent adverse prognostic factor for both EFS (HR = 3.25, 95% CI 1.75–6.03; *p* < 0.001) and OS (HR = 3.31, 95% CI 1.59–6.87; *p* = 0.001). Furthermore, age ≥ 65 years was a risk factor for inferior OS (HR = 2.20, 95% CI 1.37–3.53; *p* < 0.001), whereas the FAB-M2 subtype predicted better EFS (HR = 0.47, 95% CI 0.23–0.98; *p* = 0.044) and OS (HR = 0.39, 95% CI 0.18–0.83; *p* = 0.015).

EFS, event-free survival; OS, overall survival; VA, venetoclax plus azacitidine; HR, hazard ratio; CI, confidence interval; ELN, European LeukemiaNet; FAB, French-American-British classification; ECOG, Eastern Cooperative Oncology Group.

### Allo-HSCT Outcomes and Exploratory *KIT* Inhibition

The decision to proceed with allo-HSCT was based on a composite assessment of post-remission genetic risk (ELN 2022), MRD status, donor availability, and the patient’s age and comorbidities. To evaluate the impact of allo-HSCT on survival, we performed a time-dependent Cox analysis to account for immortal time bias, which revealed that allo-HSCT was associated with significantly improved EFS (HR = 0.09, 95% CI 0.01–0.62, *p* = 0.015) and OS (HR = 0.11, 95% CI 0.02–0.77, *p* = 0.027) (Table S3, Online Resource 1). Concurrently, we conducted an exploratory analysis of avapritinib in nine patients with KIT-mutated AML. Given its potential role in eradicating MRD, bridging refractory patients to transplant, or as post-transplant maintenance, avapritinib was employed in these contexts. The cohort predominantly consisted of CBF-AML (7/9 patients). Notably, two patients achieved rapid CR with concurrent MRD and KIT mutation negativity after only one cycle of avapritinib combined with VA. Furthermore, three with refractory AML attained CRi following avapritinib and successfully proceeded to allo-HSCT, subsequently receiving avapritinib maintenance with favorable tolerability.

### *KIT* Mutation Clearance and Relapse

 The *KIT* mutation clearance rate at 12 months was similar between the exon 17 and non-exon 17 mutation groups (60.0% vs. 57.1%; Figure S6, Online Resource 1). However, molecular relapse occurred in 51.9% (27/52) of patients who achieved initial remission. Notably, exon 17 mutations dominated both cases of molecular relapse and persistent positivity, comprising 84.6% (11/13) of the latter.

## Discussion

This study provides evidence for the prognostic significance of KIT mutations in shaping first-line treatment outcomes in AML. Our analysis suggests that IC is associated with superior outcomes compared to VA in KIT-mutated patients; that KIT mutation is an independent adverse prognostic factor for resistance to VA; and that exon 17 mutations define a subgroup with particularly poor outcomes. Furthermore, we identify allo-HSCT as an effective strategy to overcome the poor prognosis associated with *KIT* mutations in VA-treated patients. These findings position *KIT* mutation status as a valuable biomarker for guiding initial therapy selection in AML.

Although *KIT* mutations are well-established as a high-risk factor in CBF-AML [2, 7, 8, 11], their prognostic significance in non-CBF-AML—particularly in the context of the VA regimen—has remained unclear. Our findings support the notion that KIT mutation is an important biomarker of primary resistance to first-line VA therapy. This is corroborated and extended by our multivariate analysis, which confirmed it as an independent risk factor for both EFS and OS, thereby extending its adverse prognostic impact to the broader population of AML patients receiving low-intensity chemotherapy. Although survival differences were not statistically significant after PSM, likely due to limited sample size, the robust results from the multivariate analysis, which fully adjusted for confounding variables, strongly support the independent predictive value of *KIT* mutations.

The inferior efficacy of the VA regimen in *KIT*-mutated AML is likely mediated by the constitutive activation of the *KIT* signaling pathway, which enhances cell proliferation and survival [12, 13]. Specifically, *KIT* receptor mutations (notably D816V) cause ligand-independent activation of its tyrosine kinase domain, leading to persistent signaling through downstream pathways such as STAT5, PI3K/AKT/mTOR, and RAS/MAPK [9, 14, 15]. These pathways are key upstream regulators of MCL-1 expression [16, 17]. Given that MCL-1 overexpression is a well-established core mechanism of resistance to venetoclax [18, 19], its upregulation becomes critical: MCL-1 functionally complements BCL-2, so that even when venetoclax inhibits BCL-2, high levels of MCL-1 can bind and sequester pro-apoptotic proteins like BIM. This prevents the activation of BAX/BAK, maintains mitochondrial membrane integrity, and ultimately inhibits apoptosis [20–24]. Therefore, *KIT* mutation likely confers resistance to the VA regimen by driving MCL-1 expression [25]. This mechanism also explains the superior efficacy of IC observed in our study, as IC can rapidly clear leukemic clones independent of this specific anti-apoptotic pathway. Collectively, while the survival advantage of IC over VA in KIT-mutated AML was attenuated after rigorous adjustment for confounding via PSM—a common consequence of reduced sample size—the consistent direction of effect, superior numerical outcomes, and the compelling multivariate evidence position KIT mutation as a marker of resistance to VA. For medically fit patients with KIT-mutated AML, these data support the prioritization of IC as a first-line treatment option.

Within *KIT*-mutated AML, molecular heterogeneity was linked to distinct clinical outcomes. Patients with exon 17 mutations exhibited inferior EFS. Structurally, exon 17 encodes the activation loop, and mutations in this region (e.g., D816V) induce conformational changes that lead to potent constitutive *KIT* activation [26–30], providing a molecular basis for the enhanced survival advantage of the leukemic cells and the consequent aggressive clinical behavior of this subtype. *KIT* clearance kinetics further revealed unique behavior: although the 12-month clearance rate was similar to that of the non-exon 17 group, over half (51.9%) of all patients achieving molecular remission experienced *KIT* mutation reemergence, which was predominantly driven by exon 17-mutated clones. This suggests that exon 17 mutations not only contribute to initial treatment resistance but may also enhance clonal regenerative capacity and molecular relapse risk. Thus, dynamic molecular monitoring and effective maintenance strategies are particularly important for these patients.

Co-mutation analysis identified that concurrent *NRAS* mutations were associated with improved survival in *KIT*-mutated patients—a finding that contrasts with the traditional adverse prognostic role of *NRAS* in AML. This may be explained by the genetic context of our cohort, which included a substantial proportion of CBF-AML, a subtype where *NRAS* mutations have been previously reported to lack adverse or even exhibit neutral prognostic effects [31, 32]. In contrast, *FLT3-ITD/TKD* co-mutations showed a trend toward poorer outcomes. This is consistent with the known biology of *FLT3* mutations, which drive leukemogenesis through the constitutive activation of multiple downstream signaling pathways, including STAT5, PI3K/AKT, and RAS/MAPK, leading to synergistic effects on cell proliferation, survival, and inhibition of apoptosis [33–35].

Given the poor response to VA and high risk of molecular relapse observed in *KIT*-mutated patients, the finding that allo-HSCT emerged as an independent protective factor in this context strongly supports its consideration as a key consolidation strategy for this high-risk subgroup.

Although our exploratory analysis of nine patients receiving the *KIT* inhibitor avapritinib [36–38] did not demonstrate a significant survival benefit, it enabled rapid treatment responses and successfully bridged refractory patients to transplant. These clinical observations support its further evaluation in larger cohorts.

The interpretation of our findings must be considered in the context of the study’s limitations. Firstly, the sample size of the KIT-mutated/VA cohort was relatively small (*n* = 17), which limits the statistical power of subgroup analyses and may explain why the survival differences became non-significant after PSM due to further sample reduction. Therefore, the observed superiority of IC over VA, particularly in the matched analysis, should be interpreted as a strong trend requiring validation. Secondly, its retrospective, single-center design may introduce selection bias. Additionally, although treatment strategies followed clinical standards, variations in specific drug dosing and treatment cycles existed; such real-world heterogeneity might introduce unmeasured confounding factors. Future multicenter, prospective studies with larger sample sizes are essential to validate these findings, explore targeted and combination therapies for KIT-mutated AML, and further elucidate the molecular mechanisms of treatment resistance in this patient subset.

## Conclusion

This study suggests that KIT mutation status holds dual significance in guiding first-line treatment for newly diagnosed AML. Our findings indicate that KIT mutations, particularly the exon 17 subtype, are associated with poor response to the VA regimen and a high risk of molecular relapse, thereby supporting the consideration of IC as a preferred treatment option for eligible patients. Collectively, these results indicate that KIT mutation status may provide a crucial basis for individualized treatment decision-making in AML, though validation in prospective studies is warranted.

## Supplementary Information

Below is the link to the electronic supplementary material.

Supplementary Material 1

## Data Availability

Data are available from the corresponding author upon reasonable request.
