# Supplementary material for: Superior response and survival of intensive chemotherapy over venetoclax plus azacitidine in newly diagnosed KIT-mutated acute myeloid leukemia
Source: Ann Hematol. 2026 Feb 16;105(4):119. doi: 10.1007/s00277-026-06841-4 (PMC12909349; doi:10.1007/s00277-026-06841-4)
Supplement: Supplementary file 1 — Supplementary Material 1 [file 277_2026_6841_MOESM1_ESM.pdf]

Supplementary information for

Superior Response and Survival of Intensive Chemotherapy Over Venetoclax Plus Azacitidine in Newly Diagnosed KIT-Mutated Acute Myeloid Leukemia

Qingli Ji[1], Xinwen Jiang[1], Xiaoqing Li[1], Chen Cao[1], Xinrui Zhang[1], Minran Zhou[1], Sai Ma[1], Chunyan Chen[1,*]

[1]Department of Hematology, Qilu Hospital, Shandong University, Jinan, China

Annals of Hematology

*** Correspondence to:**

Prof. Chunyan Chen

Department of Hematology, Qilu Hospital, Shandong University, No. 107 Wenhuaxi Road, Jinan, 250012, China

E-mail: chency@sdu.edu.cn

Supplementary Material

Index

A. List of Abbreviations ……………………………………..3

B. Supplementary Table ....………………………………………..................4-6

Table S1. Composition of the Targeted Next-Generation Sequencing Panel

Table S2. Spectrum and Distribution of KIT Mutations in 67 Patients with KIT-Mutated AML

Table S3. Impact of allo-HSCT on Survival in VA-Treated Patients

C. Supplementary Figures ....……………………………………………7-12

Figure S1. Survival After PSM: IC vs VA in KIT-Mutated AML

Figure S2. Survival After PSM: KIT-Mutated vs Wild-Type in VA-Treated Patients

Figure S3. Impact of NRAS Co-mutations in KIT-Mutated AML

Figure S4. Impact of FLT3-ITD/TKD Co-mutations in KIT-Mutated AML

Figure S5. Multivariate Analysis of Prognostic Factors in VA-Treated Patients based on ELN 2024 risk classification

Figure S6. KIT Mutation Clearance at 3, 6, and 12 Months by Mutation Subgroup

A. List of Abbreviations

AML	acute myeloid leukemia
IC	Intensive Chemotherapy
VA	Venetoclax plus Azacitidine
EFS	Event-Free Survival
OS	Overall Survival
CR	Complete Remission
ORR	Overall Response Rate
MRD	Minimal Residual Disease
PSM	Propensity Score Matching
ELN	European LeukemiaNet
FAB	French-American-British
ECOG	Eastern Cooperative Oncology Group
HR	Hazard Ratio
CI	Confidence Interval
allo-HSCT	Allogeneic Hematopoietic Stem Cell Transplantation

B. Supplementary Table

Table S1. Composition of the Targeted Next-Generation Sequencing Panel.

DNA Sequencing Genes (n=72)

ANKRD26	ABCB1	ARID1A	ARID1B	ARID2	ASXL1	ASXL2	ATG2B
BCOR	BCORL1	BRAF	CALR	CBL	CEBPA	CREBBP	CSF3R
DDX41	DIS3	DNMT3A	ETNK1	ETV6	EZH2	FLT3	GATA1
GATA2	GFI1	GNB1	GSKIP	HRAS	IDH1	IDH2	IKZF1
JAK1	JAK2	JAK3	KDM6A	KIT	KMT2A	KMT2B	KMT2C
KRAS	MPL	MYC	NBN	NF1	NPM1	NRAS	NTRK1
NT5C2	PHF6	PPM1D	PTPN11	RAD21	RARA	RUNX1	SBDS
SETBP1	SETD2	SF3B1	SMC1A	SMC3	SRSF2	STAG2	STAT5A
TERC	TERT	TET1	TET2	TP53	U2AF1	WT1	ZRSR2

RNA Fusion Genes (n=54)

ABL1	ABL2	AFDN	BCOR	BCR	CBFA2T3	CBFB	CSF1R
DEK	EPOR	EPS15	ETV6	FGFR1	FIP1L1	FLT3	FOX04
GATA2	HOXA11	HOXA13	HOXA9	HOXA11	HOXA13	KIF5B	KMT2A
LYN	MECOM	MLF1	MLLT1	MLLT10	MLLT11	MLLT3	MLLT6
MRTFA	MYH11	NPM1	NSD1	NUMA1	NUP214	NUP98	PDGFRA
PDGFRB	PML	PRKAR1A	RARA	RBM15	RUNX1	RUNX1T1	SEPTIN6
SEPTIN9	SET	STAT5B	STRN	TBL1XR1	ZBTB16		

Table S2. Spectrum and Distribution of KIT Mutations in 67 Patients with KIT-Mutated AML

KIT mutation	Number of Mutation Events	Percentage (%)
Exon 17	53	79.10
D816V	35	66.04
D816Y	5	9.43
D816H	3	5.66
N822K	17	32.08
Other exon 17	1	1.89
Exon 8	14	20.90
Other exons	5	7.46

This table details the distribution of specific KIT mutations identified in the study cohort of 67 KIT-mutated AML patients. As some patients harbored multiple KIT mutations, the total number of mutational events exceeds the number of patients. Mutations are categorized by exon location, with exon 17 mutations further subdivided by specific amino acid changes. The D816V variant was the most prevalent. Percentages for individual exon 17 mutations are calculated relative to the total number of patients with exon 17 mutations (n=53), while all other percentages are calculated relative to the total KIT-mutated cohort (n=67).

Table S3. Impact of allo-HSCT on Survival in VA-Treated Patients

A. Time-Dependent Cox Regression Analysis

Outcome	HR (95% CI)	P-value
OS	0.11 (0.02–0.77)	0.027
EFS	0.09 (0.01–0.62)	0.015

B. Landmark Sensitivity Analysis

Landmark Time (months)	P-value	Total Patients	Transplanted Patients
4	0.589	146	1
6	0.089	137	9
8	0.022	121	13

C. Cohort Characteristics

Characteristic	Number of Patients
Total cohort	172
Transplanted	14
Non-transplanted	158
6-month landmark cohort	137

Analysis of allo-HSCT impact using time-dependent Cox regression to account for immortal time bias. The time-dependent analysis treated transplantation as a time-varying covariate. Landmark analyses were performed as sensitivity analyses at multiple time points.

C. Supplementary Figures

Figure S1. Survival After PSM: IC vs VA in KIT-Mutated AML

(a) EFS in the matched cohorts.

(b) OS in the matched cohorts.

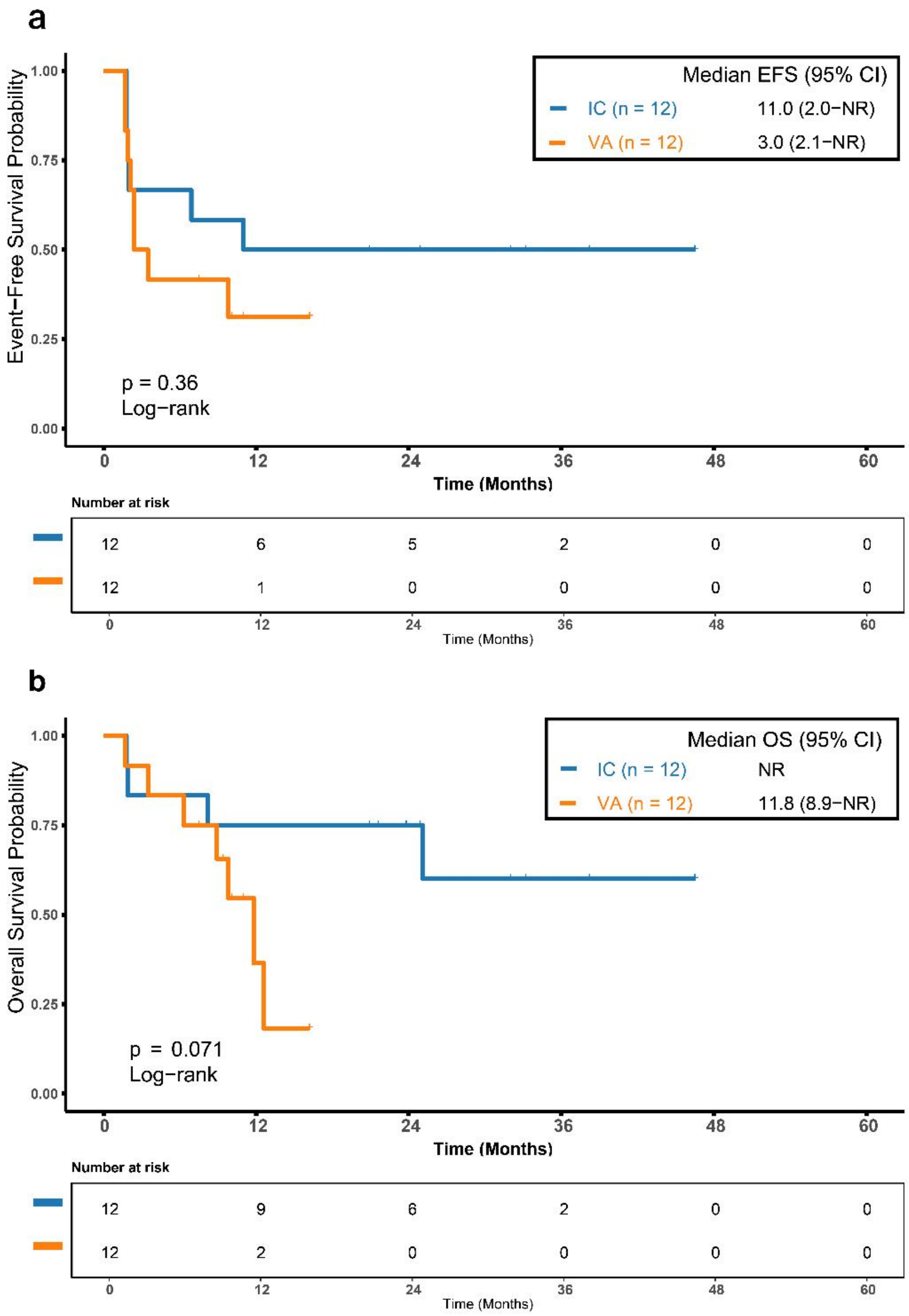

After PSM (12 pairs), the intensive chemotherapy cohort maintained numerical advantages in median EFS (11.0 vs 3.0 months, p=0.36) and median OS (not reached vs 11.8 months, p=0.071), although these differences were not statistically significant.

Figure S2. Survival After PSM: KIT-Mutated vs Wild-Type in VA-Treated Patients

(a) EFS in the matched cohorts.

(b) OS in the matched cohorts.

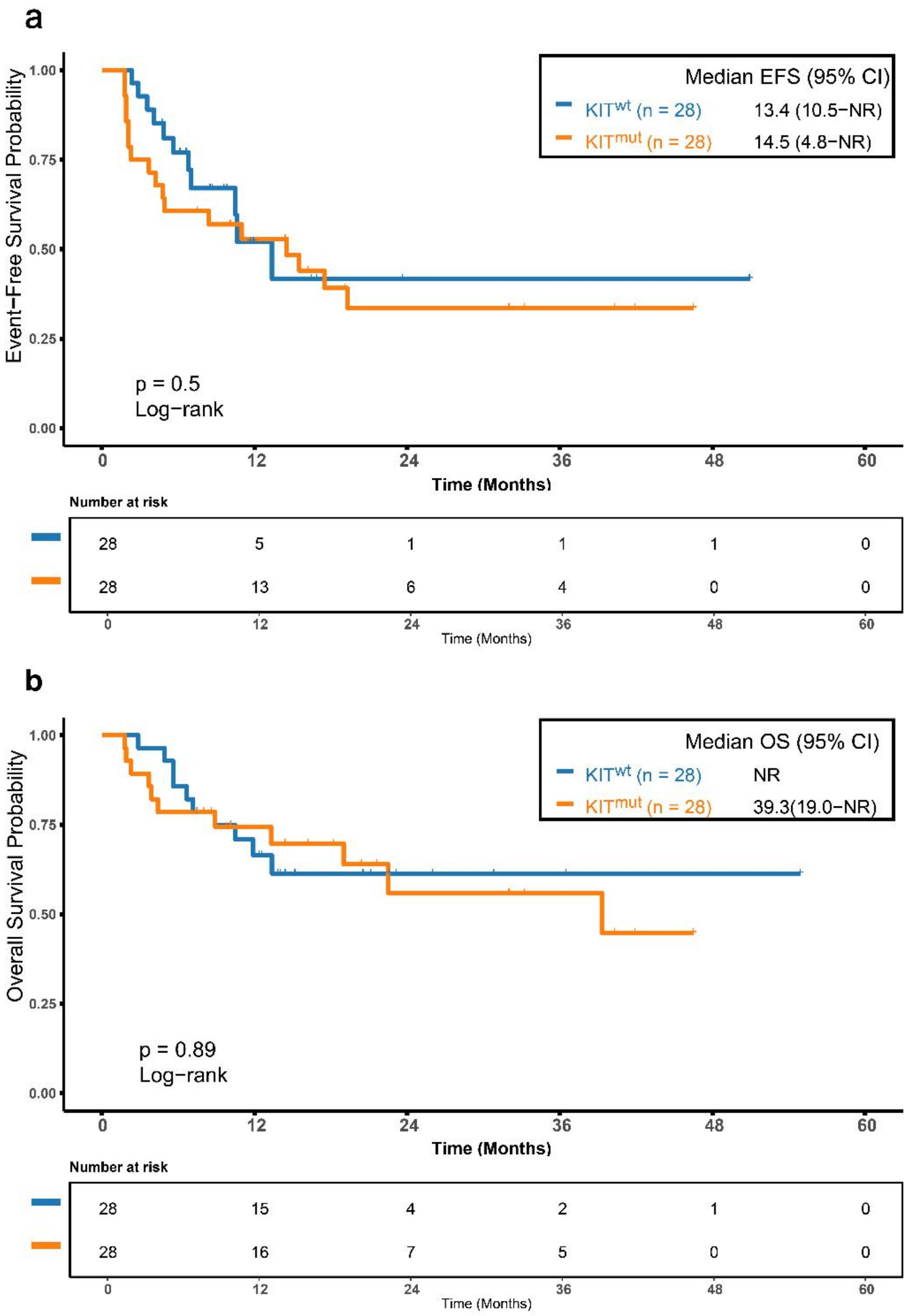

After PSM (28 pairs), differences in EFS (14.5 vs 13.4 months, p=0.5) and OS (39.3 months vs not reached, p=0.89) between KIT-mutated and wild-type patients were no longer statistically significant.

Figure S3. Impact of NRAS Co-mutations in KIT-Mutated AML

(a) EFS by NRAS mutation status.

(b) OS by NRAS mutation status.

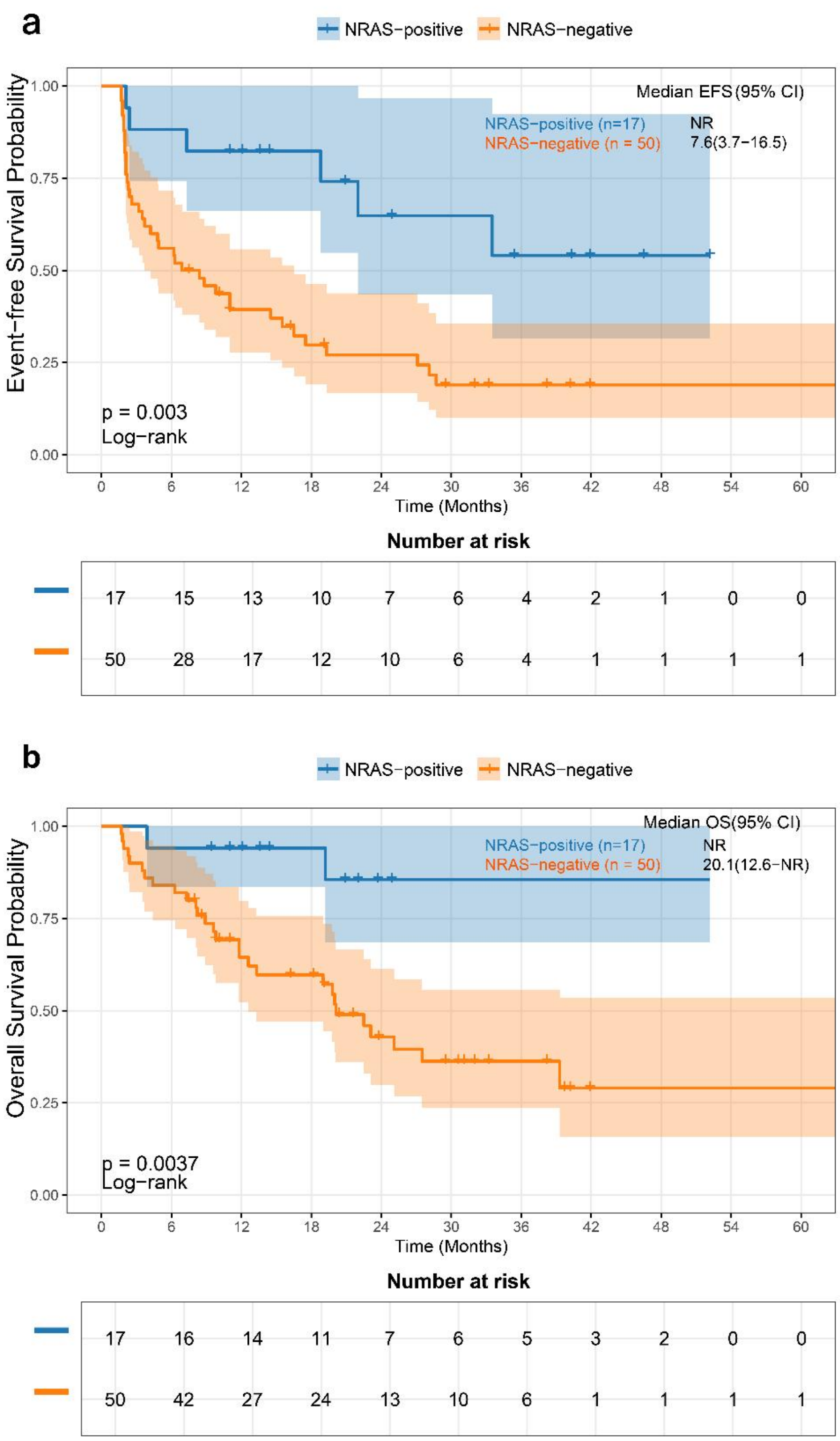

KIT-mutated patients with concurrent NRAS mutations had significantly longer EFS (not reached vs 7.6 months, p=0.003) and OS (not reached vs 20.1 months, p=0.0037) compared to NRAS wild-type patients.

Figure S4. Impact of FLT3-ITD/TKD Co-mutations in KIT-Mutated AML

(a) EFS by FLT3-ITD/TKD mutation status.

(b) OS by FLT3-ITD/TKD mutation status.

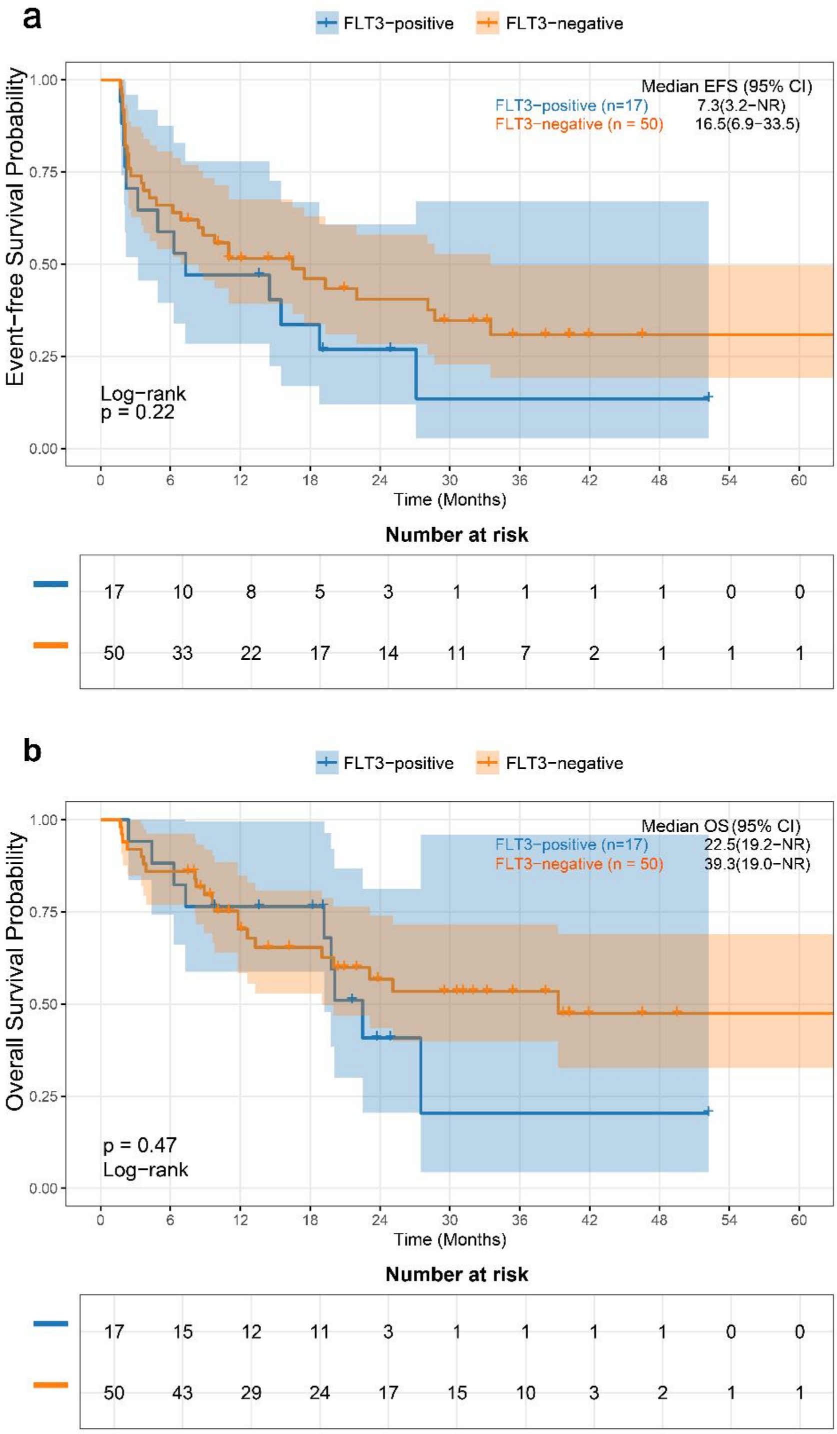

KIT-mutated patients with FLT3-ITD/TKD mutations showed a trend toward inferior median EFS (7.3 vs 16.5 months, p=0.22) and median OS (22.5 vs 39.3 months, p=0.47) compared to FLT3 wild-type patients, though these differences were not statistically significant.

Figure S5. Multivariate Analysis of Prognostic Factors in VA-Treated Patients based on ELN 2024 risk classification.

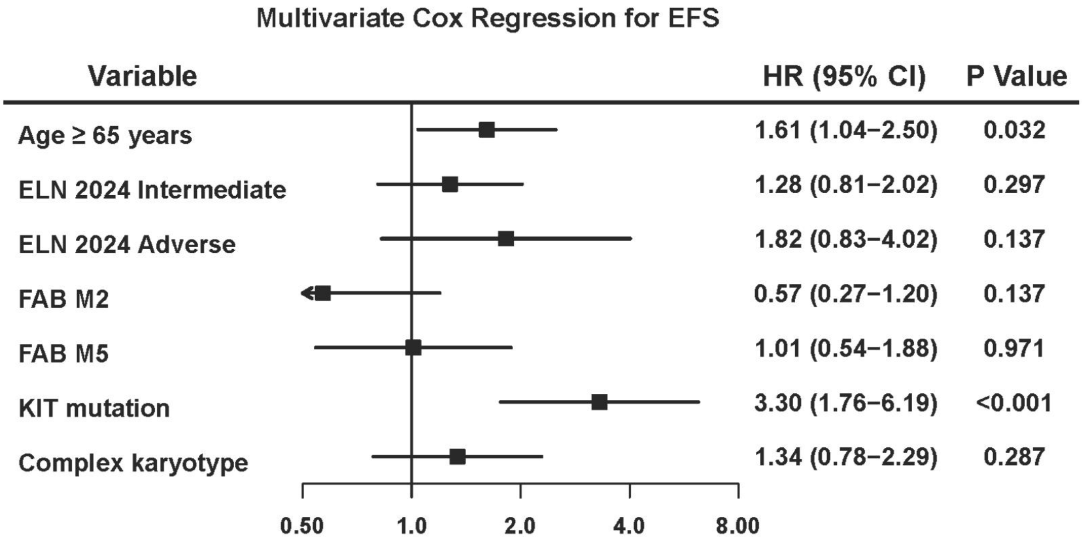

(a)

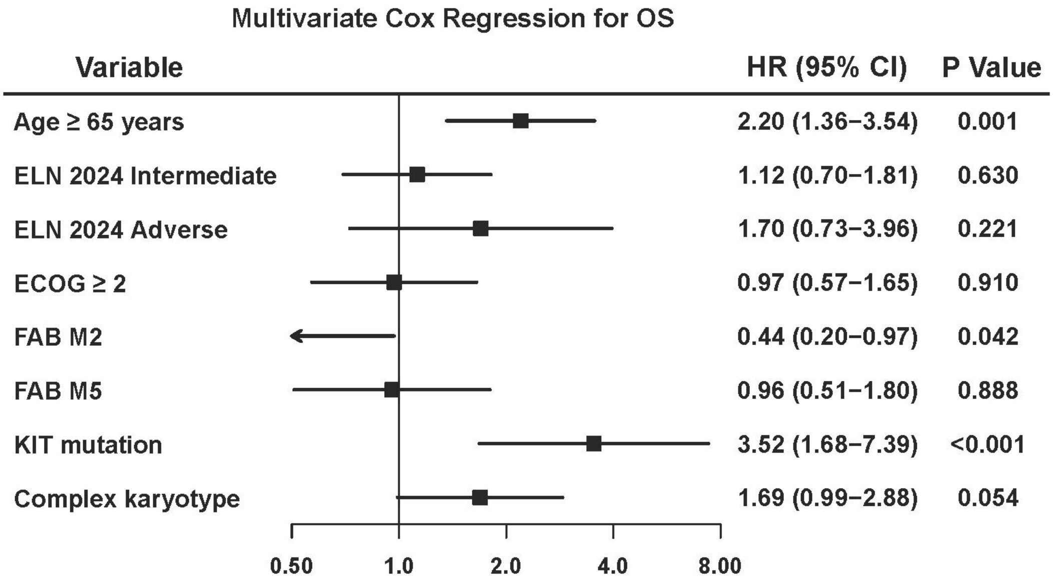

(b)

Figure S6. KIT Mutation Clearance Rates at 3, 6, and 12 Months by Mutation Subgroup

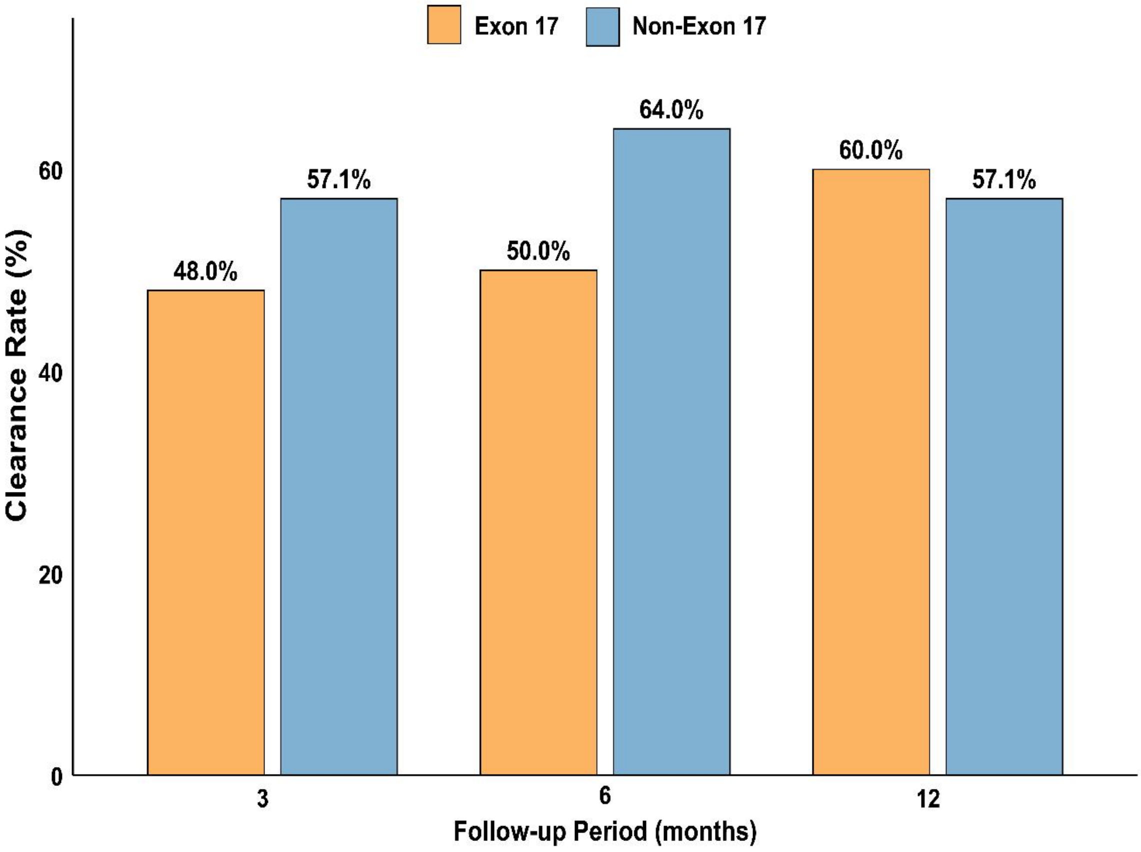

Bar graph comparing the rates of KIT mutation clearance, as assessed by next-generation sequencing, in patients with exon 17 mutations versus non-exon 17 mutations at 3, 6, and 12 months after treatment initiation
